# Voltage gated sodium and calcium channels: Discovery, structure, function, and Pharmacology

**DOI:** 10.1080/19336950.2023.2281714

**Published:** 2023-11-20

**Authors:** William A. Catterall

**Affiliations:** Department of Pharmacology, University of Washington, Seattle, WA, USA

**Keywords:** sodium channel, calcium channel, protein structure, cryogenic electron microscopy, X-ray crystallography

## Abstract

Voltage-gated sodium channels initiate action potentials in nerve and muscle, and voltage-gated calcium channels couple depolarization of the plasma membrane to intracellular events such as secretion, contraction, synaptic transmission, and gene expression. In this Review and Perspective article, I summarize early work that led to identification, purification, functional reconstitution, and determination of the amino acid sequence of the protein subunits of sodium and calcium channels and showed that their pore-forming subunits are closely related. Decades of study by antibody mapping, site-directed mutagenesis, and electrophysiological recording led to detailed two-dimensional structure-function maps of the amino acid residues involved in voltage-dependent activation and inactivation, ion permeation and selectivity, and pharmacological modulation. Most recently, high-resolution three-dimensional structure determination by X-ray crystallography and cryogenic electron microscopy has revealed the structural basis for sodium and calcium channel function and pharmacological modulation at the atomic level. These studies now define the chemical basis for electrical signaling and provide templates for future development of new therapeutic agents for a range of neurological and cardiovascular diseases.

## Sodium channels initiate action potentials

Voltage-gated sodium (Na_V_) channels are found in both kingdoms of prokaryotes and throughout the four kingdoms of eukaryotes. In the majority of these physiological settings, sodium channels generate conducted action potentials in response to small membrane depolarizations. In their classic work on sodium channels in nerve axons, Hodgkin and Huxley showed that small membrane depolarizations activate voltage-gated sodium channels, which generate action potentials that are conducted along the length of the nerve axons of the squid [1]. Sodium channels play a similar role in skeletal muscle and cardiac muscle fibers [2,3]. In addition, in endocrine cells and many other cell types that are not cylindrical, sodium channels initiate action potentials that are conducted over the cell body [4].

## Calcium channels couple membrane depolarization to calcium entry

In some excitable cells, such as Paramecium and invertebrate skeletal muscle, voltage-gated calcium channels initiate and conduct calcium-dependent action potentials similarly to sodium channels [5,6]. Moreover, in repetitively firing cells of the sinoatrial node in the heart and the thalamus in the brain, calcium channels participate in rhythmic firing of action potentials [7–9]. However, the primary role of voltage-gated calcium channels is to couple depolarization of the cell surface membrane to calcium entry that initiates and regulates intracellular events such as contraction, secretion, neurotransmission, and gene expression [10–13]. Compared to sodium channels, the physiological roles of calcium channels are more varied and multifaceted, and their structures and functions are similarly more diverse.

## Sodium and calcium channels as toxin targets

Voltage-gated sodium and calcium channels are the molecular targets for a broad array of natural toxins that are used in defense and in attacking prey [14,15]. They are produced, stored, and released by species ranging from single-celled dinoflagellates and corals to complex organisms such as spiders, frogs, and snakes. All of these chemical agents take advantage of the essential role of sodium and calcium channels in nerve conduction and synaptic transmission to stun, immobilize, and kill prey and predators, typically by binding specifically with high affinity to target receptor sites in the ion channel structure. These naturally occurring ion channel ligands act as gating modifiers by altering voltage-dependent gating or as pore-blockers by physically occluding the pore [14,15].

## Finding sodium channels

**Figure 1.**
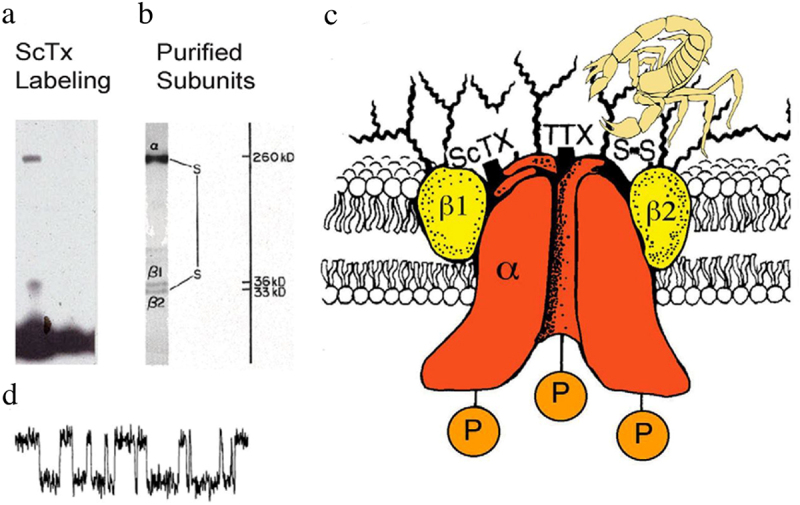
Subunit structure of voltage-gated sodium channels. a) SDS polyacrylamide gel electrophoresis illustrating the α and β subunits of rat brain sodium channels covalently labeled with^125^I-labeled *leiurus quinquestriatus* scorpion toxin (ScTx) and imaged by autoradiography. Adapted from Beneski and Catterall, 1980 [16]. b) SDS polyacrylamide gel electrophoresis patterns illustrating the α and β subunits of the brain Na^+^ channels. Sodium channel purified from rat brain showing the α, β1, and β2 subunits and their molecular weights. Adapted from Hartshorne et al., 1982 [19]. As illustrated, the α and β2 subunits are linked by a disulfide bond. Tetrodotoxin (TTX) and scorpion toxins (ScTx) bind to the α subunits of Na^+^ channels as indicated and were used as molecular tags to identify and purify the sodium channel protein from brain. c) drawing of the subunit structure of the brain Na^+^ channel based on biochemical data. Ψ, sites of N-linked glycosylation. Adapted from Catterall, 1984 [27]. d) single channel currents conducted by a single purified Na^+^ channel incorporated into a planar bilayer [23].

Discovery of the sodium channel protein was directly dependent upon use of neurotoxins that bind to them with high affinity as molecular probes. α-Scorpion gating-modifier toxins from *Leiurus quinquestriatus* were used to photoaffinity label the protein subunits of sodium channels in mammalian brain with a photoreactive arylazide toxin derivative (Figure 1(a) [16]). This method identified two protein subunits, a large α-subunit with a molecular weight of 260 kilodaltons (kDa) and a smaller β-subunit of 33–36 kDa [17]. A complex of the large α subunit with two closely related β1 and β2 subunits was solubilized and purified from rat brain using specific binding of the pore blocker saxitoxin as a molecular probe (Figure 1(b) [18–20]) and shown to function as a voltage-gated sodium channel when reconstituted into phospholipid vesicles (Figure 1(c) [21,22]) or planar phospholipid bilayers (Figure 1(d)) [23]. All three subunits were found to be hydrophobic membrane glycoproteins. A similarly large, highly glycosylated sodium channel α subunit was identified in purified sodium channel preparations from electric eel electroplax and mammalian skeletal muscle [24–26]. These results led to formulation of a biochemical model for the subunit structure of sodium channels (Figure 1(c) [27]).

**Figure 2.**
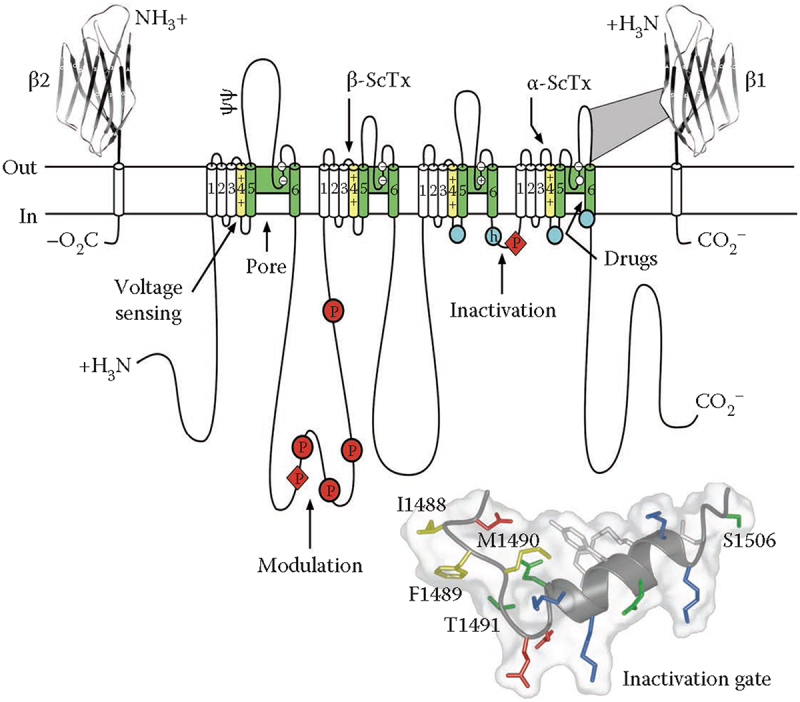
The primary structures of the subunits of the voltage-gated sodium channels. Cylinders represent alpha helical segments. Bold lines represent the polypeptide chains of each subunit with length approximately proportional to the number of amino acid residues in the brain sodium channel subtypes. The extracellular domains of the β1 and β2 subunits are shown as immunoglobulin-like folds. Ψ, sites of N-linked glycosylation; P in red circles, sites of demonstrated protein phosphorylation by PKA (circles) and PKC (diamonds); green, pore-lining segments; white circles, the outer and inner (DEKA) rings of amino residues that form the ion selectivity filter and the tetrodotoxin binding site; yellow, S4 voltage sensors; h in blue circle, inactivation particle in the inactivation gate loop; blue circles, sites implicated in forming the inactivation gate receptor. Sites of binding of α- and β-scorpion toxins and a site of interaction between α and β1 subunits are also shown. Tetrodotoxin is a specific blocker of the pore of Na^+^ channels, whereas the α- and β-scorpion toxins block fast inactivation and enhance activation, respectively, and thereby generate persistent Na^+^ current that causes hyperexcitability and depolarization block of nerve conduction. Adapted from Catterall, 2000 [34]. *Inset*. Structure of the fast inactivation gate in solution determined by NMR. Adapted from Rohl et al. [86].

A key advance in studies of sodium channels was cloning and sequencing cDNA encoding the α subunits based on amino acid sequence and antibodies derived from biochemical studies of purified sodium channels – first from electric eel electroplax [28] and then from mammalian brain [29–31], skeletal muscle [32], and heart [33]. These results provided the key insights into the primary structures of sodium channels, which were used in extensive structure-function studies for more than three decades (reviewed in [34–36]). Some of the highlights of these studies are summarized in the form of a transmembrane folding model for the sodium channel (Figure 2). The ~ 2000 amino acid residues of the sodium channel α subunit are arranged in 24 transmembrane segments, which are organized in four homologous domains, DI to DIV (Figure 2). The six transmembrane segments in each domain were numbered S1-S6. The amino acid sequence is approximately 50% identical in the conserved transmembrane domains, but the large intracellular loops that connect the four homologous domains differ greatly from each other. The intracellular linker connecting Domains III and IV was found to serve as the inactivation gate (Figure 2, inset). The smaller β1 and β2 subunits were also cloned and sequenced using primary structure information and antibodies derived from biochemical studies [37,38]. These subunits are closely related single membrane-spanning cell adhesion molecules having a large extracellular N-terminal domain with an immunoglobulin-like folding pattern and a short intracellular C-terminal domain [39]. Related β3 and β4 subunits were cloned and analyzed many years later [40,41].

## Finding calcium channels

**Figure 3.**
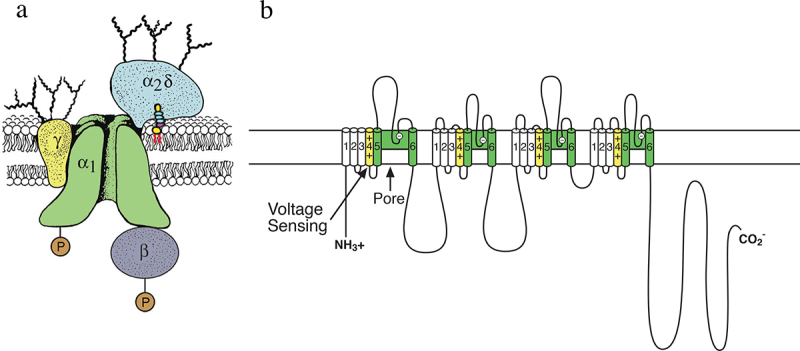
The subunit structure of calcium channels purified from skeletal muscle. a) a biochemical model of the skeletal muscle calcium channel taken from the original description of the subunit structure of skeletal muscle Ca^2+^ channels but with the mature α2δ subunit depicted following proteolytic processing, disulfide bond formation and attachment of a glycosylphosphatidylinositol membrane anchor. Adapted from Takahashi et al., 1987 [48]. P, sites of phosphorylation by cAMP-dependent protein kinase and protein kinase C. Ψ, sites of N-linked glycosylation. b) transmembrane folding models of the Ca_V_1.1 subunits. Predicted alpha helices are depicted as cylinders. The lengths of lines correspond approximately to the lengths of the polypeptide segments represented. Adapted from Catterall, 1991 [49].

The protein subunits of calcium channels were discovered using similar ligand binding and biochemical purification methods as for sodium channels, but the primary ligands were radiolabeled calcium antagonist drugs in the dihydropyridine family, including nifedipine and isradipine [42,43]. Initial purification studies revealed α, β, and γ subunits from skeletal muscle, an abundant calcium channel protein source [44–47]. However, more detailed biochemical analysis, covalent-labeling, lectin-binding studies, and subunit-specific antibodies led to a surprisingly complex subunit structure with five protein subunits organized as in Figure 3(a) [48,50,51]. The large transmembrane α1 subunit was found to be associated with an intracellular transmembrane β subunit, a glycosylated transmembrane γ subunit, and a glycosylated transmembrane complex of disulfide-linked α2 and δ subunits [48,52–55]. This protein complex was functional in voltage-gated calcium conductance when reconstituted into phospholipid vesicles and planar phospholipid bilayers [45,47,56].

Cloning and sequencing the cDNA encoding the large α1 subunit of the skeletal muscle calcium channel revealed a protein of 2000 amino acid residues, closely resembling the sodium channel α subunit in transmembrane architecture, but only 25% identical in amino acid sequence (Figure 3(b) [57]). Further cloning and sequencing studies confirmed that the β subunit is an intracellular subunit, while the γ subunit has four transmembrane segments and multiple glycosylation sites, as expected from biochemical data [53,54]. Surprisingly, cloning, sequencing, and biochemical studies showed that the disulfide-linked α2 and δ subunits are encoded by a single gene [58], proteolytically processed to yield the disulfide-linked α2 and δ polypeptides [59,60], and further processed to cleave and replace the apparent transmembrane segment of the δ subunit with a glycosylphosphatidylinositol membrane anchor (Figure 3(a)) [61]). These complex processing and protein interactions of the α2δ subunits may be involved in regulation of synaptic plasticity [62].

## Structure/Function studies of sodium and calcium channels

The structure and function of sodium and calcium channels have been studied extensively by site-directed mutagenesis and by mapping with site-directed antibodies (reviewed in [34–36]). Some of the highlights of these extensive studies are presented in the following sections.

### Voltage dependent activation

In their classic work on voltage clamp analysis of sodium channels in the squid giant axon, published in 1952 long before any molecular studies of sodium channels, Hodgkin and Huxley proposed based on physical principles that voltage-dependent activation must involve the outward movement of three positively charged “gating particles” across the membrane upon depolarization, which led to opening of sodium channels [1]. This prophetic proposal has guided most subsequent work on the mechanism of activation of sodium channels. The predicted outward movement of the gating particles, now called “gating charges,” was detected by high-resolution voltage clamp studies of the squid giant axon in the absence of sodium and other permeant ions [63,64]. Armstrong and Bezanilla detected tiny capacitative “gating currents” produced by outward movement of the gating charges upon sodium channel activation and their subsequent inward movement upon repolarization and deactivation [63,64]. Detailed measurements with more modern methods have detected the movement of 12 to 16 gating charges per sodium channel voltage sensor [65,66]. But where are the gating charges located and how do they work? The S4 segments in each domain contain four to seven repeats of a highly conserved three-residue motif of a positively charged amino acid residue (usually Arg) flanked by two hydrophobic residues [35,67]. This structure creates a ladder of positive charges across the membrane. The “*sliding-helix*” model of voltage sensing [67–69] posited that the positively charged residues in the S4 segment were neutralized and stabilized in their transmembrane position by ion pair interactions with negatively charged amino acid residues in the neighboring transmembrane segments. In the resting state, these positive charges are pulled toward the cytosol by the negative membrane potential. Upon depolarization, this electrostatic force is released, and the S4 gating charges move outward along a spiral pathway by exchanging ion pair partners [67–69]. In support of this mechanism, mutations that neutralize the gating charges alter the voltage dependence of activation [70–73], toxin labeling studies show that the S4 segment remains in a transmembrane position in both resting and activated states [74,75], and substituted Cys labeling and crosslinking experiments show that the gating charges do indeed exchange ion pair partners and become accessible at the cell surface [76–78]. A consensus article by several leading investigators supports the sliding-helix model of voltage sensing [79], and the structural basis for the sliding helix model of voltage sensing and activation has now been further established as described below.

### Fast inactivation

As shown by Hodgkin and Huxley [1], sodium channels inactivate within a few milliseconds after opening. Fast inactivation can be prevented by proteolytic treatment of the internal compartment of the squid axon, placing the inactivation process on the intracellular surface of the membrane [80]. Studies with sequence-direct antibodies targeted to each of the intracellular linkers showed that the short, highly conserved linker between Domains III and IV of the α subunit can impair fast inactivation of the sodium current and can induce re-openings of single sodium channels due to block of fast inactivation (Figure 2 [81,82]). Similarly, expression of the sodium channel as two separate proteins cut between Domains III and IV prevents fast inactivation [70]. Mutations in a conserved hydrophobic motif (Ile-Phe-Met, IFM) in the linker between Domains III and IV can completely prevent fast inactivation [83], which can be restored by intracellular perfusion of a short peptide containing the IFM motif (KIFMK [84,85]). The structure of the inactivation particle was first elucidated by NMR analysis of the intracellular linker between Domains III and IV expressed as a separate protein (Figure 2, inset [86]). Altogether, these studies led to a model of fast inactivation in which the IFM motif folds into the intracellular surface of the channel and inhibits ion conductance through the pore [83].

### Slow inactivation

Voltage-gated sodium channels have a second, much slower inactivation process [87], which is engaged in tens to hundreds of milliseconds and regulates action potential generation during long trains of impulses [88]. It is not affected by treatment of the intracellular surface of the channel with proteases, suggesting that conformational changes in the transmembrane part of the sodium channel protein may be involved [89]. Extensive mutagenesis studies indicate that slow inactivation involves conformational changes in the outer half of the pore, involving the S5 and S6 segments as well as their extracellular connecting loops [90–92].

### Ion Conductance and selectivity

The high affinity neurotoxins tetrodotoxin and saxitoxin block sodium channels from the extracellular solution in a manner that is consistent with direct occlusion of the pore [4]. The first evidence for the location of the pore came from identification of the amino acid residues in the P loop connecting the extracellular ends of the S5 and S6 segments as components of the receptor site for the pore blocker tetrodotoxin [93]. Mutagenesis studies revealed that sodium channels could be converted to calcium selectivity by mutation of a single amino acid residue in each of the four P loops to the negatively charged residue Glu, placing these residues in position to form the ion selectivity filter [94]. These studies led to a model in which the extracellular end of the pore of the sodium channel is lined by the P loops in Domains I -IV, which are surrounded by the S5 and S6 transmembrane segments [95].

## Three dimensional structure of an ancestor of sodium and calcium channels

**Figure 4.**
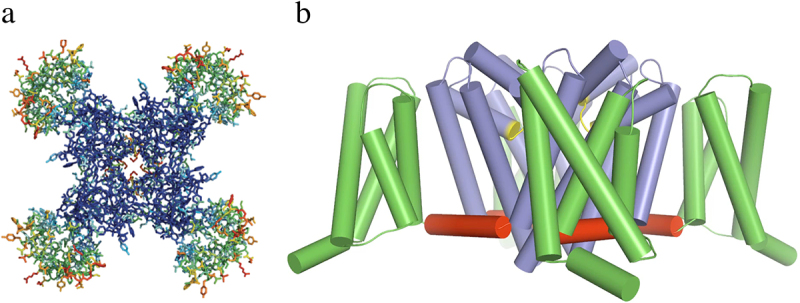
Structure of the bacterial sodium channel Na_V_Ab. a) top view of Na_V_Ab channels colored according to crystallographic temperature factors of the main-chain (blue <50 Å^2^ to red > 150 Å^2^). The four pore modules in the center are rigid in the crystal structure and therefore are blue. The four voltage-sensing modules surround the pore and are more mobile, as illustrated by warmer colors. b) side view of Na_V_Ab. Voltage sensing module (S1-S4), green; pore module (S5, S6, and P loop), blue; selectivity filter, yellow; S4-S5 linker, red. Adapted from Payandeh et al.,2011 [97].

The α subunits of sodium channels are among the largest and most hydrophobic membrane proteins, which greatly slowed their study by structural biology methods. However, the unexpected discovery of prokaryotic voltage-gated sodium channels [96] provided an experimental model that allowed structural studies by X-ray crystallography. These prokaryotic channels are formed from a homotetramer of subunits that are similar in structure to one domain of a mammalian sodium channel, and biochemical and structural studies are facilitated by the lack of large intracellular and extracellular linkers [96]. The voltage-gated sodium channel from *Arcobacter butzleri* is a protein of 285 amino acid residues that can be expressed at high levels in insect cells driven by the powerful baculovirus promoter [97]. This channel protein could be expressed, purified, and crystallized in good yield and analyzed by X-ray crystallography [97]. The resulting structure at a resolution of 2.7 Å revealed the three-dimensional structure of a voltage-gated sodium channel for the first time [97]. As viewed from the extracellular side, the central pore is surrounded by four pore-forming domains composed of the S5 and S6 segments and the P loops between them (Figure 4(a), blue). Four voltage-sensing modules composed of the S1-S4 transmembrane segments are located on the periphery of the structure in a nearly symmetrical square array (Figure 4(a), green). As viewed from the membrane side, the S4-S5 linkers in each domain are seen as alpha-helical segments lying along the inner surface of the membrane (Figure 4(b), red). The four domains are linked in a domain-swapped organization, such that each voltage-sensing module is covalently connected to the pore module of its neighbor [97]. This organization may enforce concerted activation and opening of all four subunits simultaneously, thereby contributing in an important way to the rapid rate of increase of sodium current and the beginning of an action potential.

**Figure 5.**
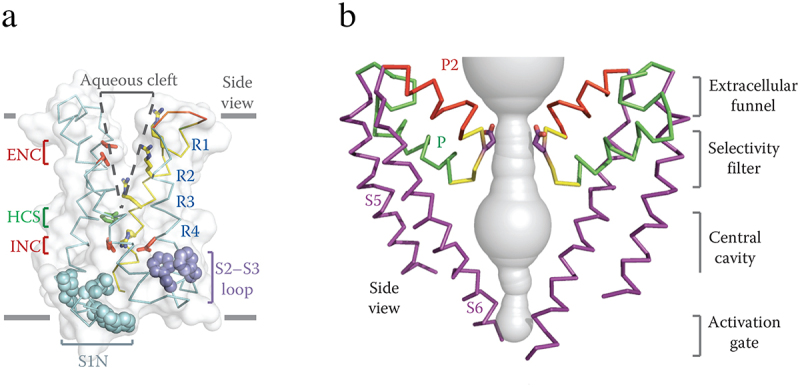
Architecture of the Na_V_Ab voltage sensing module and pore module. a) side view of the voltage-sensing module of Na_V_Ab illustrating the conformations of the S1-S4 helices and the size of the extracellular aqueous cleft with the R1-R4 gating charges (blue), extracellular negative cluster (ENC, red), intracellular negative cluster (INC, red), and hydrophobic constriction site (HCS, green). b) side view of the pore-forming module of two subunits of Na_V_Ab. S5 and S6 transmembrane helices, purple: P helix loop, green; P2 helix green; water-filled space revealed by MOLE, gray. Note that the two S6 segments are crossed at their intracellular ends, forming the closed conformation of the activation gate. Adapted from Payandeh et al., 2011 [97].

The original structure of Na_V_Ab revealed the overall design of the voltage sensor and pore module (Figure 5 [97]). The voltage sensor is a V-shaped array of the S1-S4 segments, with S1-S2 forming one helical pair and S3-S4 forming a second helical pair. The two helical bundles are separated by an extracellular aqueous cleft that penetrates approximately half of the depth of the membrane (Figure 5(a) [97]). The key Arg gating charges, labeled R1-R4, are arrayed across the membrane in the S4 segment. The positively charged side chains of R1-R3 protrude into the extracellular aqueous cleft, and they make ion pair interactions with the negatively charged side chains of Glu residues in the Extracellular Negative Cluster (ENC; Figure 5(a), red [97]). In contrast, the positively charged side chain of R4 is located below the inner end of the extracellular aqueous cleft and makes interactions with the negatively charged side chains of the Intracellular Negative Cluster (INC, red). In between the ENC and INC is a hydrophobic barrier, the Hydrophobic Constriction Site (HCS, green), which seals the voltage sensor against penetration by water and ions. The structure of the voltage sensor suggests that is designed to move the gating charges in the S4 segment outward and inward in response to changes in the electrical field and trigger conformational changes to open and close the pore [97]. The voltage sensors of Na_V_Ab were in an activated conformation in this structure.

The pore is formed by the S5 and S6 segments and the connecting P loop (Figure 5(b)). As a sodium ion approaches the pore from the extracellular solution, it enters a wide vestibule followed by the narrow ion selectivity filter. It then exits into the large water-filled central cavity and eventually moves into the cytosol through the activation gate formed by the intracellular ends of the four S6 segments (Figure 5(b)). In this first structure of Na_V_Ab, the pore was in the closed conformation, with the four S6 segments binding tightly to each other. Remarkably, sodium ions complete this transit through the open pore at a rate of ~ 10^7^ per second.

## Structural basis for voltage dependent activation and pore opening in Na_V_Ab

**Figure 6.**
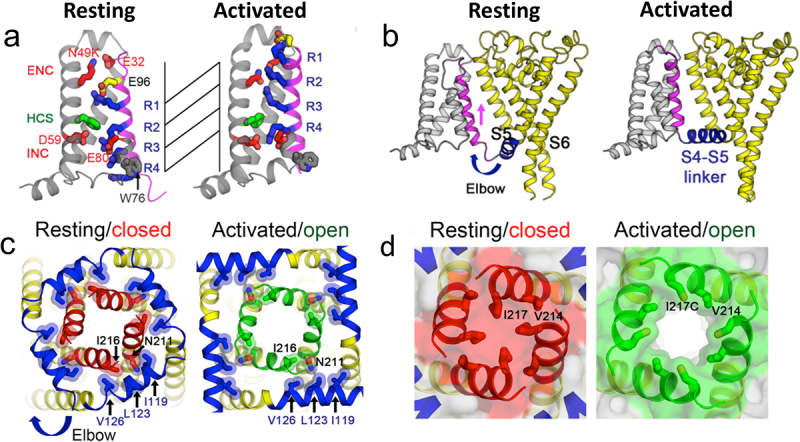
Sodium channel activation and pore gating mechanism of Na_V_Ab. a) gating charge movement. Four Arg gating charges, R1–R4 (blue); the extracellular negative charge (ENC) cluster of E32 and N49(K) and the intracellular negative charge (INC) cluster of E59 and E80 (red); Phe in the hydrophobic constriction site (HCS) (green); and conserved W76 (gray) and E96 (yellow) are shown as sticks. S4 (magenta) moves outward by 11.5 Å, passing two gating charges through the HCS. Part of S3 is omitted for clarity. b) sideview of the structures focusing on S4 (magenta) and the S4-S5 linker (blue), with the S0–S3 segments shown in gray and the pore module in yellow. The S4 segment moves outward across the membrane from the resting to the activated states, whereas the S1–S3 segments remain relatively unchanged with respect to the membrane. The S4-S5 linker acts as an elbow that connects the S4 movement to modulate the pore. c) bottom (intracellular) view of the structures in (b), with S0–S4 omitted for clarity. The S4-S5 linker (blue) undergoes a large conformational change that tightens the collar around the S5 (yellow) and S6 segments (red or green) of the PM in the resting state and loosens the collar in the activated state. d) space-filling model of the structures in (c) at high magnification. Adapted from Wisedchaisri et al., 2019 [98].

The structural basis for activation of the voltage sensor was revealed by comparison of high-resolution structures of Na_V_Ab in resting and activated states [97,98]. The resting state was captured by introducing mutations that positively shifted the voltage dependence of activation by 250 mV, which stabilized the resting state at 0 mV, and by forming a disulfide bond between two substituted Cys residues to lock the voltage sensor in its resting conformation. Successful trapping of the functional resting state was confirmed by reducing the disulfide bonds, releasing the voltage sensor from its inactivated trapped position, and allowing it to respond to membrane potential with full recovery of sodium channel function [98]. The structures of Na_V_Ab in resting and pre-open states are very similar, but there is a remarkable conformational change in the voltage sensor (Figure 6 [98]). When viewed from the side, the S4 segment is pulled toward the cytosol by 11.5 Å, which results in positioning the R2-R4 gating charges on the intracellular side of the HCS (Figure 6(a) [98]). This movement forms an elbow at the intracellular end of S4 where it joins the S4-S5 linker (Figure 6(b) [98]). In essence, this striking conformational change captures the energy of the electric field in the inward elbow conformation of the S4-S5 linker. This conformation serves as a cocked gun, ready to shoot the gating charges outward upon depolarization of the membrane (Figure 6(b) [98]). Looking outward toward the intracellular side of the voltage sensor, the four S4-S5 linkers form a nearly square corral (Figure 6(c), blue) surrounding the intracellular ends of the S6 segments (Figure 6(c), left, red). In the activated state, the four S4-S5 linkers move ~ 45° in a clockwise direction (Figure 6(c), blue). In contrast, the movement of the S6 helical backbone of the S6 segments is much more subtle (Figure 6(c), left, red/green). However, a space-filling representation shows a dramatic change. In the resting state, the orifice of the activation gate formed by the intracellular ends of the S6 segments is completely closed by the side chains of the four I217 residues (Figure 6(d), left, red shading), whereas these side chains are rotated out of the orifice of the pore in the open state leaving a pathway of 10.5 Å diameter for ion permeation (Figure 6(d), right, white). This orifice is sufficient for rapid permeation of hydrated sodium ions, as illustrated in the Movies in Reference [98].

## Structural analysis of ion conductance and selectivity in Na_V_Ab

**Figure 7.**
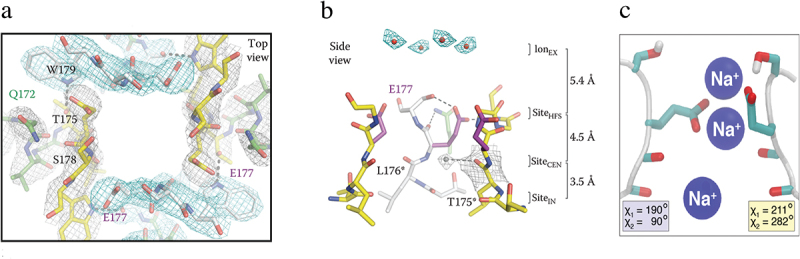
Mechanism of sodium conductance and selectivity. a) top view of the ion selectivity filter illustrating the high field strength site formed by four E177 residues. Hydrogen bonds between T175 and W179 are indicated by gray dashes. b) side view of the ion selectivity filter. E177 (purple) interactions with Q172, S178 and the backbone of S180 are shown for one subunit; putative cations or water molecules (red spheres). Electron-density around L176 (gray) and a bound water molecule are shown in gray mesh. Na^+^-coordination sites: Site_HFS_, Site_CEN_ and Site_IN_. Adapted from (Payandeh et al. 2011) [97]. c) E177 dunking. Movement of Na^+^ through the ion selectivity filter catalyzed by inward movement (dunking) of the side chains of E177 via a single torsion angle bend. Adapted from Chakrabarti et al., 2013 [99].

As a sodium ion approaches the outer edge of the ion selectivity filter, it passes through the high field-strength site (HFS) formed by the side chains of four negatively charged Glu residues (E177, Figure 7(a) [97]). Interaction with the high field-strength site requires the sodium ion to release some of its waters of hydration, which are replaced by Glu side chains as ligands. The sodium ion then moves through the central and inner ion binding sites, which are formed by backbone carbonyls of L176 and T175 (Figure 7(b) [97]). Although the helical backbone that forms the walls of the ion selectivity filter is rigid, molecular dynamics analysis shows that the side chains of E177 “dunk” with the sodium ion and catalyze its inward movement (Figure 7(c) [99]). This rapid dunking movement requires only rotation around a single torsion angle, allowing it to take place on the time scale of ion conductance–10^7^ ions/sec.

## Multi-phase slow inactivation and drug block in Na_V_Ab at the atomic level

**Figure 8.**
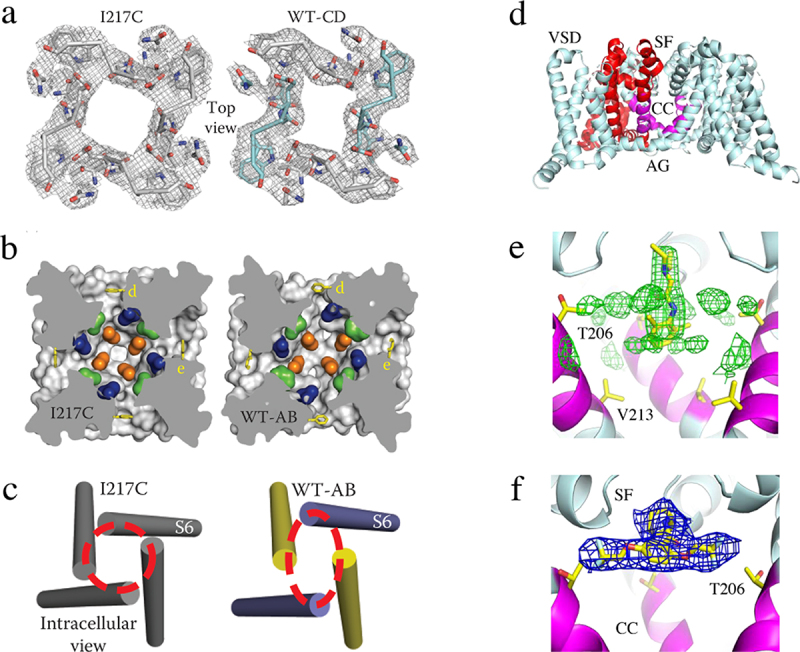
Slow inactivation and drug block of Na^+^ channels. a) top view of the collapse of the pore during slow inactivation of Na_V_Ab. Two S6 segments move inward the central axis of the pore and two move outward to produce an asymmetric, partially collapsed conformation. The selectivity filter structure has changed from nearly square in the pre-open state of Na_V_Ab/I217C to a partially collapsed parallelogram in the inactivated state of Na_V_Ab/WT-CD. b) the central cavity is partially collapsed. c) the activation gate is tightly closed, but collapsed into a two-fold symmetric conformation. Adapted from Payandeh et al, 2012 [102]. d) structure of Na_V_Ab with the selectivity filter (SF), central cavity (CC), and activation gate (AG) highlighted. e) lidocaine bound in the central cavity at the base of the selectivity filter. f) flecainide bound at the local anesthetic/antiarrhythmic receptor site in the central cavity at the base of the selectivity Filter.

Homotetrameric prokaryotic sodium channels do not have the structural equivalent of the fast inactivation gate of metazoan sodium channels, but they do have a process similar to slow inactivation that is mediated by the pore module [100]. Structure-function studies show that Na_V_Ab has three kinetic phases of inactivation, which result in a highly stable inactivated state [101]. The structure of this stable inactivated state was determined by X-ray crystallography [102]. The overall structure is very similar to Na_V_Ab in the pre-open state, but there are striking conformational changes in the pore [102]. Two of the S6 segments that line the pore have moved toward the central axis and the other two have moved away, resulting in a structure with two-fold symmetry rather than four-fold symmetry (Figure 8 [102]). The ion selectivity filter is in the shape of a parallelogram, rather than square (Figure 8(a)). A similar conformational change is observed in the central cavity (Figure 8(b)). Finally, the intracellular ends of the S6 segments where they form the activation gate have an oval configuration rather than circular (Figure 8(c)). Extensive structure-function studies implicate both the outer pore and the S6 segments in slow inactivation, consistent with this structural change [91,92,103].

Local anesthetics and Class I antiarrhythmic drugs inhibit sodium channels in a frequency- and voltage-dependent manner, which allows them to preferentially reduce sodium current and action potential generation in rapidly firing neurons that signal pain and in damaged cardiomyocytes that generate arrhythmias [104,105]. Both the local anesthetic lidocaine and the antiarrhythmic drug flecainide bind in the central cavity of Na_V_Ab in a position to block sodium exit from the ion selectivity filter (Figure 8(d–f) [106]). Remarkably, access to this receptor site is controlled both by entry through the intracellular mouth of the pore in the open state and through fenestrations in the sides of the pore that lead to the into the central cavity from lipid bilayer in the resting state [106]. The rate of drug entry through the pore in the transient open state compared to the entry through fenestrations in the long-lasting resting state determines the extent of voltage- and frequency-dependent block of the pore, which is a critical element of controlling drug action [106].

## Structural basis for calcium selectivity

**Figure 9.**
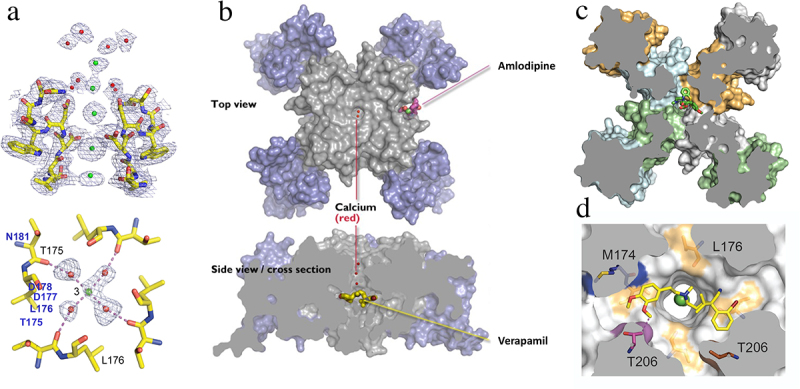
Calcium and drugs binding to the pore module of Ca_V_Ab. a) side view of the ion selectivity filter of Ca_V_Ab from X-ray crystallography. a) the ion selectivity filter of Ca_V_Ab at high resolution. Green balls, calcium ions; red balls, water; mesh, electron density. Top. side view; bottom, bottom view illustrating a single calcium ion with a square array of four waters of hydration bound. Adapted from Tang et al., 2014 [113]. b) structure of Ca_V_Ab in top and side views by X-ray crystallography. Blue, voltage sensor; gray, pore module. Bound amlodipine and verapamil, yellow sticks. c) top view of a cross-section of Ca_V_Ab with diltiazem bound in its receptor site as indicated (green sticks). Adapted from Tang et al., 2018 [138]. d) bottom X-ray crystallographic view of a cross-section in high resolution with verapamil (yellow sticks) bound in its receptor site and a calcium ion (green) bound in the pore. Adapted from Tang et al., 2014 [113].

The prokaryotic sodium channel Na_V_Ab is a member of a large protein family whose members are as similar in amino acid sequence to mammalian calcium channels as to sodium channels and likely served as evolutionary precursors to both sodium and calcium channels [107,108]. These ancestral channels provide an exceptional opportunity to mimic evolution and build calcium channel features in their ancestral setting, which is amenable to high-resolution structural studies. Calcium channels must conduct calcium rapidly and selectively, yet the tight calcium binding needed for high selectivity would block the pore and prevent high conductance. Biophysical models resolved this paradox by combining high affinity binding to multiple calcium binding sites in sequence in the pore in order to induce electrical repulsion between bound calcium ions [109–112]. In this situation, calcium binds tightly in the pore and prevents other ions from permeating, but approaching calcium ions can “knock off” resident calcium ions in the pore and generate high conductance. Substitution of only three amino acid residues in the vestibule and selectivity filter of Na_V_Ab is sufficient to mimic vertebrate calcium channel structure and form Ca_V_Ab, a calcium channel construct whose calcium selectivity is comparable to mammalian cardiac calcium channels with P_Ca_/P_Na_ ~400 (Figure 9(a), top [113]). Structural analysis shows that these amino acid substitutions do not alter the backbone fold of the voltage sensor ([113]); however, high-resolution views from X-ray crystallographic analysis reveals a series of calcium binding sites (Figure 9(a), green balls with mesh) that lead from the outer vestibule through to the central cavity. In favorable samples, one layer of water molecules is observed between the bound calcium ion and the walls of the selectivity filter (Figure 9(a), bottom [113]). The spacing of these calcium binding sites is ideal for electrostatic repulsion (Figure 9(a) [113]), which would knock off the resident calcium ion and allow rapid conductance. Consistent with this model, binding of a single blocking divalent cation, such as cadmium or nickel, would generate pore block rather than high conductance by producing high affinity binding without the knock-off effect [113]. These results provide the structural basis for the paradox of simultaneous high selectivity and high conductance that are essential for calcium channel function.

## Pharmacological modulation of the model calcium channel Ca_V_Ab

Voltage-gated calcium channels are the molecular targets for calcium antagonist drugs that are used in treatment of cardiac arrhythmia, hypertension, and angina pectoris [114,115]. Phenylalkylamines like verapamil and benzothiazepines like diltiazem are primarily used to treat atrial arrhythmias, whereas dihydropyridines like nifedipine and amlodipine are primarily used for treatment of hypertension and angina pectoris [116]. The sites of action of these important therapeutic agents have been progressively resolved by ligand binding, photoaffinity labeling, site-directed mutagenesis, and high-resolution structure determination, and they give key insights into the mechanisms of action of these important drugs [117–119]. Ligand binding studies showed that these three chemical classes of drugs have three allosterically coupled receptor sites, a distinct receptor site for the dihydropyridines and two separate but overlapping sites for verapamil and diltiazem [42,43]. Binding of verapamil and diltiazem and their derivatives is frequency-dependent, similar to the pore-blocking local anesthetics that inhibit sodium channels [120], suggesting that these calcium antagonist drugs are also pore-blockers [105,121]. In contrast, binding of dihydropyridines is voltage-dependent but not frequency-dependent, suggesting that they may interact allosterically with the voltage sensor rather than block the pore [105,122]. Photoaffinity labeling and antibody mapping of the labeled receptor sites showed that phenylalkylamines and benzothiazepines label the S6 segments in Domains III and IV that line the pore [123,124]. Photoaffinity labeling and antibody mapping also showed that dihydropyridines bind to the S5 and S6 segments in Domain III and the S6 segment in Domain IV [125,126]. These findings were greatly amplified by extensive site-directed mutagenesis studies of the S5 and S6 segments of Domains III and IV [127–136]. Functional dihydropyridine receptor sites were constructed by substitution of only nine amino acid residues in the IIIS5, IIIS6, and IVS6 segments, confirming that all of the important molecular interactions had been identified [133,135,136]. Altogether, this work on ligand binding, photoaffinity labeling, and site-directed mutagenesis indicated that phenylalkylamines and benzothiazepines bind to overlapping sites in the pore and block it, whereas dihydropyridines bind to a separate site on the membrane-facing aspect of the pore module and inhibit ion conductance through the pore in an indirect allosteric manner [117–119].

Structural studies have further resolved these receptor sites. In the prokaryotic model calcium channel Ca_V_Ab (Figure 9(b) [137,138]), amlodipine and other benzodiazepines bind to a receptor site on the lipid-facing surface of the pore module, between two voltage sensing domains, through interactions with the membrane-facing surface of the S5 and S6 segments (Figure 9(b) [137]). In contrast, verapamil binds to a site in the central cavity of the pore and physically occludes it (Figure 9(b) [137]). Similarly, diltiazem binds to an overlapping site and blocks the pore (Figure 9(c) [138]). High-resolution views of bound verapamil reveal that its tertiary amino group is lodged at the exit from the ion selectivity filter into the central cavity, while its two hydrophobic rings bind tightly to two sides of the pore and adhere tightly like a band-aid (Figure 9(d)). These two distinct types of receptor sites, which have been characterized at the atomic level, reveal the structural basis for frequency-dependent block of the pore by verapamil and diltiazem versus allosteric, voltage-dependent inhibition of calcium channels by dihydropyridines.

Key insights into the binding and action of these drugs have also come from high resolution cryogenic electron microscopy studies of the mammalian skeletal muscle calcium channel by Professor Nieng Yan and her colleagues [139,140], as summarized in the third article in this Special Issue. This remarkable work gives dramatic new insights into the subunit architecture, pore, and drug receptor sites of mammalian voltage-gated calcium channels at near-atomic resolution.

## Structure of the cardiac sodium channel

Studies of mammalian sodium and calcium channels have benefited greatly from the revolution in high-resolution cryo-EM (Nieng Yan, Articles 2 and 3 in this Special Issue [139,141]). In addition to the structure of the skeletal muscle Ca_V_1.1 channel noted above, these powerful methods have opened the way to new high-resolution structures of sodium channels from nerve [142–146], skeletal muscle [141], and heart [147,148], as well as calcium channels from peripheral nerve and brain [149,150]. Some highlights of the structure of the cardiac sodium channel as revealed by cryo-EM studies in our laboratory [147] are summarized here, and the structures and pharmacology of nerve and skeletal muscle sodium channels are summarized in more detail in Article 2 of this Special Issue by Professor Nieng Yan [141,142].

**Figure 10.**
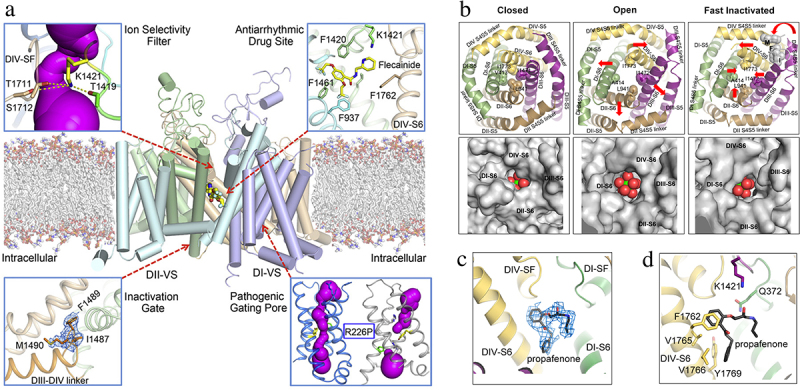
The cardiac sodium channel Na_V_1.5 at high resolution. a) graphical abstract. Center. Na_V_1.5 structure. Upper left, sodium permeation pathway in the pore module, purple. Upper right, bound flecainide. Lower right, arrhythmia mutation R227P in the voltage sensor. The pathogenic gating pore caused by this mutation creates a complete water-filled pathway through the voltage sensor, as revealed with the program MOLE (purple). Lower left. IFM motif of the fast inactivation gate. b) bottom view of the structure of the activation gate formed by the inner ends of the alpha-helical S6 segments. Top row, backbone structure in closed, open, and fast inactivated states. Bottom row. A sodium ion (yellow) with waters of hydration (red). Adapted from Jiang et al., 2020 [147]. c) propafenone binding site in side view. d) propafenone binding site in top view.

The structure of the cardiac sodium channel has been captured in both closed/inactivated and open states [147,151]. The structure of the closed/inactivated state of the cardiac sodium channel was determined using a construct in which the large intracellular linkers and C-terminal domain, which are predicted to be unstructured, were deleted by site-directed mutagenesis [152]. The resulting Na_V_1.5c construct is fully functional and retains its characteristic sensitivity to toxins and antiarrhythmic drugs [147]. The alpha-helical backbone of the transmembrane core of Na_V_1.5c (Figure 10(a), center [147]) is nearly identical to Na_V_Ab, with root mean square deviation (RMSD) of 3.2 Å, well within the level of uncertainty expected due to the limits of resolution of the cryo-EM structure (3–4 Å). The four voltage sensors are in different activated conformations with 2–4 gating charge Arg residues positioned on the extracellular side of the HCS (Figure 10(a), center [147]). The pore is closed by the intracellular activation gate formed the four S6 segments. The fast inactivation gate is tightly bound to a receptor site adjacent to the IVS6 segments and the closed activation gate (Figure 10(a), lower left [147]). Thus, this structure has all of the features expected for a closed/inactivated state of the sodium channel.

## Structural basis for pathogenic gating pores

The voltage sensors of sodium channels are tightly packed, such that the movement of the S4 segment through the hydrophobic constriction site takes place without significant leakage of water and ions (Figure 6). However, mutation of one of the Arg gating charges to a smaller, hydrophilic residue can cause pathogenic ion leakage, termed “gating pore current,” through the hydrophobic constriction site when the small amino acid side chain occupies the hydrophobic constriction site of the sodium channel without fully blocking it [153]. This persistent ionic leak is the cause of periodic paralysis [154–156], cardiac arrhythmia [157], and autism [158]. Structural studies of a pathogenic gating pore inserted in the ancestral sodium channel Na_V_Ab introduces a hole of ~ 3 Å diameter in the voltage sensor, just large enough for permeation of a sodium ion [159]. The pathogenic gating pore mutation R226P in rNa_V_1.5, which causes cardiac arrhythmia [157], is illustrated in Figure 10(a), lower right. The resulting complete water-filled pathway is illustrated in purple for the mutant, whereas the water-filled pathway is interrupted by the presence of Arg226 in wild-type (Figure 10(a), lower right). These pathogenic gating pores allow persistent leak of sodium into nerve and muscle cells, which causes persistent depolarization, increased resting membrane conductance, and impairment of action potential firing.

## Structural basis for sodium conductance and selectivity in the cardiac sodium channel

The pore of Na_V_1.5c is asymmetric and tortuous (Figure 10(a), upper left [147]). The high field-strength site of the ion selectivity filter is surrounded by four different amino acid residues – Asp-Glu-Ala-Lys (DEKA). It is surprising to find a positively charged Lys residue in a sodium-specific ion selectivity filter, but Lys in this position is absolutely required for sodium selectivity [160]. Careful analysis of the structure of the selectivity filter in Na_V_1.5c revealed that the positive charge of the ε-amino group of Lys1421 is delocalized by formation of three hydrogen bonds with uniquely placed backbone carbonyls, which points the partially negatively charged electron pair of the ε-amino group into the lumen of the pore, where it is available for interaction with conducted sodium ions [147]. These structural findings led to the *ε-electron-pair hypothesis* of sodium selectivity, which posits that this unique chemical interaction is essential for sodium selectivity [147].

## Subunit structure of the cardiac sodium channel

Vertebrate sodium channels typically have one or two Na_V_β subunits associated with their pore-forming α subunit [34,36]. However, even when the Na_V_β1 and Na_V_β2 subunits were over-expressed with Na_V_1.5c, neither subunit was resolved in the structure, suggesting that these subunits have low affinity for cardiac sodium channels. Consistent with this hypothesis, an N-linked carbohydrate chain was found in an overlapping position with the expected interaction site of the Na_V_β1 subunit, and a Cys residue that forms a disulfide linkage with the Na_V_β2 subunit was replaced by Leu [147].

## Structural basis for pore opening and fast inactivation of cardiac sodium channels

The fast Inactivation gate is formed by the short intracellular loop connecting Domains III and IV of mammalian sodium channels [70,81–83], and the hydrophobic motif Ile-Phe-Met (IFM) serves as the inactivation particle, binds to the intracellular surface of the pore module, and inhibits sodium current [83,84]. In contrast to the closed/inactivated state, Na_V_1.5c trapped in the open state by mutation of the IFM inactivation particle in the inactivation gate to the hydrophilic amino acid residues QQQ revealed a similar structure for the voltage sensors, with RMDS of 1.04 Å; however, many conformational changes were observed in the inner pore region near the activation gate and inactivation gate receptor site (Figure 10(b) [151]). In particular, the IFM motif is observed bound in its receptor site in the closed/inactivated structure, but it has dissociated and is not observed in the structure in the open state (Figure 10(b) [147,151]). The red arrows in Figure 10(b) show the motion of the amino acid residues in the activation gate in closed, open, and inactivated structures. The pore-opening mechanism is nearly identical to Na_V_Ab, but the configuration of the open activation gate is slightly asymmetric with dimensions of 10.6 Å x 9.7 Å. The open activation gate is just wide enough to accommodate a fully hydrated sodium ion (Figure 10(b) [151]), indicating that sodium conductance out of the central cavity occurs in single file, even though the total flux of sodium is very rapid at ~ 10^7^ ions/sec.

## Receptor site for antiarrhythmic drugs in the cardiac sodium channel

The antiarrhythmic drug flecainide bound in the central cavity of Na_V_1.5c, in position to block ion conductance by preventing movement of sodium ions from the selectivity filter into the central cavity (Figure 10(a), upper right [147]), as previously observed in Na_V_Ab (Figure 8(d–f) [106]). It makes prominent contacts with F937, F1420, K1421, and F1461, but the key residue F1762 is not close enough for a strong interaction (Figure 10(a), upper right [147]). In the open state of Na_V_1.5c, the antiarrhythmic drug propafenone also binds in a pore-blocking position in the central cavity (Figure 10(c,d)). Its interaction site is overlapping, yet distinct from flecainide (Figure 10(c,d) [151]). It makes a strong π-π interaction with the aromatic side chain of F1762 and additional interactions with K1421, V1760, V1765, and Y1769. These open-state interactions seem likely to provide higher affinity binding than the interactions observed for flecainide in the closed/inactivated state (Figure 10(c,d) [147,151]). Both propafenone and flecainide are Class IC antiarrhythmic drugs, which are thought to bind with high affinity to the open state of sodium channels [161,162]. Therefore, it is likely that these structures capture a low-affinity binding pose for flecainide to a closed/inactivated state (Figure 10(b)) and a high-affinity binding pose for propafenone to the open state (Figure 10(c,d)). The classical Class I antiarrhythmic drug quinidine also binds to this receptor site [148]. In addition, the atypical antiarrhythmic drug ranolazine binds to the core of the antiarrhythmic drug receptor site, but projects its atypical second aromatic ring beyond this classical drug receptor site to make new interactions with the IS6 segment that may be responsible for its unique channel-blocking properties [163]. Further studies may reveal the structural and chemical basis for state-dependent binding and block of sodium channels by these life-saving antiarrhythmic drugs.

## Receptor site for a gating modifier toxin on the cardiac sodium channel

**Figure 11.**
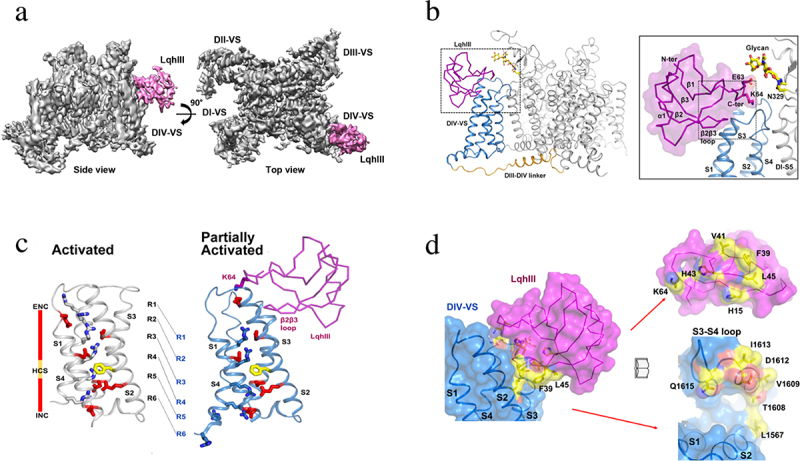
Receptor site for the deathstalker scorpion toxin LqhIII on Na_V_1.5. a) spacefilling side view and top view of Na_V_1.5 (gray) with LqhIII bound (purple). b) close-up view of bound LqhIII (purple) to its receptor site on the Na_V_1.5 backbone structure. *Inset*. higher resolution image of the interface between LqhIII and Na_V_1.5. c) superposition of the voltage sensor in the unmodified activated state and the toxin-modified partially activated state. d) mutational map of the interface residues in the LqhIII receptor site illustrated with “open book” format. LqhIII, purple; Na_V_1.5, blue. Amino acid residues highlighted in yellow and orange are required for high-affinity binding. Adapted from Jiang et al., 2021 [168].

Polypeptide gating modifier toxins bind to the extracellular surface of the voltage sensors of sodium channels and alter voltage sensor function [14,164]. For example, α-scorpion toxins bind in a voltage-dependent manner and greatly slow fast inactivation of sodium channels, leading to pathologic depolarization and repetitive firing in nerve and muscle and to life-threatening arrhythmias in the heart [164]. The receptor site for α-scorpion toxins was identified by photoaffinity labeling [16] and mapped by site-directed antibodies and site-directed mutagenesis [74,165], leading to the conclusion that these toxins bind to a receptor site formed primarily by the S3-S4 extracellular linker in Domain IV, with secondary contributions by the S1-S2 linker in Domain IV and the SS2-S6 linker in the adjacent region of Domain I. The deathstalker α-scorpion toxin LqhIII has high affinity for the cardiac sodium channel and generates lethal arrhythmias in the heart [166,167]. The structure of this toxin bound to its receptor site on cardiac sodium channels has been solved at high resolution for both closed/inactivated and open sodium channels using the Na_V_1.5c construct ([168]; Jiang et al., submitted). The toxin binds to a receptor site on the Domain IV voltage sensor (Figure 11(a)). It wedges its flexible β2-β3 loop and C-terminal region into the aqueous cleft in the voltage sensor, making strong interactions with the S1-S2 linker and S3-S4 linker (Figure 11(b) [168]), consistent with previous structure-function studies [74,165]. The voltage sensor is locked in a partially activated conformation (Figure 11(c) [168]), suggesting how binding of the toxin can allow sodium channel activation and also prevent fast inactivation at the same time. Strong interactions are observed with Asp1612 via a pincers complex with His15 and His43 (Figure 11(d)). An analogous negatively charged residue is present in this position in the binding sites of many different scorpion toxins acting on a range of sodium channel types, suggesting that this interaction is central to toxin action. The bound toxin grips an arc of amino acid residues bridging the S1-S2 and S3-S4 linkers, in position to control the movements of these two helical hairpins in the gating process (Figure 11(d)). LqhIII is bound tightly through interaction of residue K64 with the negatively charged residues of the ENC, which further stabilizes the toxin-channel complex and prevents outward movement of gating charges R1 and R2 into this position during activation of the channel (Figure 11(c,d)). This charge-charge interaction of K64 with the ENC may be responsible for the characteristic slow, voltage-dependent dissociation of LqhIII and other α-scorpion toxins, as the toxin is slowly forced off its receptor site by electrostatic repulsion from the R1 and R2 gating charges during the outward movement of the S4 segment. Altogether, these structural features explain the hallmark features of scorpion toxin action – high affinity binding to sodium channels in resting states, strong effects to prevent fast inactivation, and slow voltage-dependent dissociation.

## Conclusion

Biochemical studies in the 1980’s led to identification of the protein components of sodium and calcium channels and defined their subunit structures [27,48], and cDNA cloning and sequencing determined their primary amino acid sequences [28,29,57]. Based on these early results, extensive structure-function studies in the 1990’s and 2000’s led to key insights into the mechanisms of ion conductance and selectivity [94,160,169], voltage-dependent activation and inactivation [68,70,81,83], and pharmacological modulation [119,170]. Beginning in 2011, high-resolution structures of ancestral sodium channels [97] followed by mammalian sodium and calcium channels [139,171] have provided increasingly precise views of their mechanisms of action at atomic resolution. We look forward to deeper understanding of the fundamental mechanisms of action of these ion channels and to development of higher affinity, more efficacious, and safer drugs for use as local anesthetics, analgesics, and antiarrhythmics in the future.

## Data Availability

Because this is a review article, no new experimental data are presented. Interested individuals should write to the authors of the papers that are cited in each figure legend to obtain the data presented in that figure.
